# Field-Deployable Recombinase Polymerase Amplification Assay for Specific, Sensitive and Rapid Detection of the US Select Agent and Toxigenic Bacterium, *Rathayibacter toxicus*

**DOI:** 10.3390/biology10070620

**Published:** 2021-07-03

**Authors:** Mohammad Arif, Grethel Y. Busot, Rachel Mann, Brendan Rodoni, James P. Stack

**Affiliations:** 1 Department of Plant and Environmental Protection Sciences, University of Hawaii at Manoa, Honolulu, HI 96822, USA; arif@hawaii.edu; 2 Department of Plant Pathology, Kansas State University, Manhattan, KS 66506, USA; gybusot@gmail.com; 3 Plant Biosecurity Cooperative Research Centre, Canberra, Australia; Rachel.Mann@ecodev.vic.gov.au (R.M.); brendan.rodoni@agriculture.vic.gov.au (B.R.); 4 Inari Agricultural Inc., One Kendall Square, Cambridge, MA 02139, USA; 5 Department of Economic Development, Jobs, Transport and Resources, Biosciences Research Division, Bundoora, VIC 3083, Australia; 6 School of Applied Systems Biology, La Trobe University, Bundoora, VIC 3086, Australia

**Keywords:** biosecurity, diagnostics, isothermal amplification, field-deployable detection

## Abstract

**Simple Summary:**

Early, accurate, and rapid detection of *R. toxicus* is extremely important to improve inspections of imported annual ryegrass hay and seed at ports of entry and enhance in-field detection. RPA is a comparatively new, easy to use, and robust technology that can be performed in the palm of the hand without losing specificity. The RPA assay was more sensitive than endpoint PCR and did not require lab equipment in the field. The developed assay has tremendous applications for in-field plant diagnostics and biosecurity surveillance.

**Abstract:**

*Rathayibacter toxicus* is a toxigenic bacterial pathogen of several grass species and is responsible for massive livestock deaths in Australia and South Africa. Due to concern for animal health and livestock industries, it was designated a U.S. Select Agent. A rapid, accurate, and sensitive in-field detection method was designed to assist biosecurity surveillance surveys and to support export certification of annual ryegrass hay and seed. Complete genomes from all known *R. toxicus* populations were explored, unique diagnostic sequences identified, and target-specific primers and a probe for recombinase polymerase amplification (RPA) and endpoint PCR were designed. The RPA reaction ran at 37 °C and a lateral flow device (LFD) was used to visualize the amplified products. To enhance reliability and accuracy, primers and probes were also designed to detect portions of host ITS regions. RPA assay specificity and sensitivity were compared to endpoint PCR using appropriate inclusivity and exclusivity panels. The RPA assay sensitivity (10 fg) was 10 times more sensitive than endpoint PCR with and without a host DNA background. In comparative tests, the RPA assay was unaffected by plant-derived amplification inhibitors, unlike the LAMP and end-point PCR assays. In-field validation of the RPA assay at multiple sites in South Australia confirmed the efficiency, specificity, and applicability of the RPA assay. The RPA assay will support disease management and evidence-based in-field biosecurity decisions.

## 1. Introduction

Annual ryegrass toxicity (ARGT) is a fatal disease of livestock caused by a toxin produced by the plant-pathogenic bacterium *Rathayibacter toxicus* [1,2,3,4,5]. Outbreaks of lethal toxicoses in livestock and horses that graze on infected plants have been reported in South Australia and Western Australia for over fifty years, with hundreds to thousands of animal deaths in each outbreak [1,6]. *Rathayibacter toxicus* may have been introduced into South Africa in ryegrass hay or seed from Australia; lethal toxicoses in South Africa resulted, affecting sheep in the original outbreaks in 1980s and horses more recently, from 2009 to the present [6,7]. The *R. toxicus* bacterium is vectored by nematode species in the genus *Anguina*; *Anguina funesta* is the primary vector for *R. toxicus* in annual ryegrass (*Lolium rigidum*) [8,9,10]. *Rathayibacter toxicus* causes gummosis diseases on annual ryegrass and other grass hosts in the Poaceae family [1]. The presence of plant host species and favorable climatic conditions in the United States suggest that *R. toxicus* is an animal health and economic threat to vulnerable U.S. livestock industries and, hence, was designated a U.S. Select Agent (https://www.selectagents.gov/sat/list.htm, accessed on 17 May 2021).

Early detection and accurate identification of the pathogen is a prerequisite for preventing dissemination to other geographical regions within and/or among states/countries. Recently, Yasuhara-Bell and Stack [11] developed a LAMP assay matrix to discriminate all five populations of *R. toxicus*, to be used in conjunction with a generic LAMP assay developed by Arif et al. [12]. Luster et al. [13] also develop immunoreagents for detection of *R. toxicus*. At present, there is no validated field-deployable detection method for *R. toxicus* to support biosecurity surveillance, export certification, and outbreak response. Arif et al. [14] developed multiplex TaqMan and endpoint PCR assays to specifically detect and discriminate *R. toxicus*. However, polymerase chain reaction (PCR)-based methods require sophisticated and expensive equipment and are not practical for field application [15]. Recent advancements in isothermal amplification technologies have provided pathogen detection tools with high sensitivity and rapid assay results—ideal attributes for applications in biosecurity and point-of-care diagnostics [16]. Numerous isothermal methods, including LAMP [17], strand displacement amplification (SDA) [18], helicase-dependent amplification (HDA) [19], nicking enzyme amplification reaction (NEAR) [20], rolling circle amplification (RCA) [21], and recombinase polymerase amplification (RPA) [22], are available. LAMP is the most popular among these isothermal methods [23,24] but has some drawbacks, including complex primer design and a high reaction temperature (65 °C) requirement [25]. In recent years, RPA has become more popular because of its low sensitivity to PCR inhibitors commonly found in environmental matrices (e.g., plant tissues), high target signal sensitivity, and moderate temperature (37–39 °C) requirement for amplification [26,27]. Importantly, results can be visualized on lateral flow devices (LFDs) in less than five minutes [26,27]. Unlike LAMP, RPA protocols only require two long primers (forward and reverse) of approximately 32–35 bp in length and a probe of approximately 48–52 bp. Recently, a multiplex RPA assay, coupled with LFDs, was developed by Larrea-Sarmiento and co-workers [16] for the specific detection of *Clavibacter* species, widely prevalent Gram-positive plant-pathogenic bacteria. They too demonstrated that RPA reactions can be performed in a closed-hand palm without the need for lab equipment. RPA reactions have high target specificity and sensitivity, low sensitivity to amplification inhibitors, and rapid generation of the target amplicon within 10 min [16].

Interpretation of diagnostic assay results is dependent upon inclusion of appropriate positive and negative (non-template) controls; confidence in the conclusions drawn from those results can be substantially enhanced by the inclusion of internal controls in each reaction tube [14,28]. For in-field detection of plant-associated microbes, the addition of controls that target the host genome enhance confidence in the DNA preparation procedures [26], especially when dealing with plant tissues containing high concentrations of amplification inhibitors [29]. The objective of this research was to develop a genome-informed, reliable, sensitive, and accurate RPA assay to specifically detect the U.S. Select Agent bacterium, *R. toxicus*, from pure cultures in the lab and from infected plant materials in the field to support routine diagnostics, biosecurity surveillance, disease outbreak response, and epidemiology research.

## 2. Materials and Methods

### 2.1. Source of Cultures, Infected Plant Samples, and DNA Isolation

*Rathayibacter toxicus* strains were isolated from infected annual ryegrass samples collected from South Australia and Western Australia in 2013 and 2014 [30] (Table 1). Some strains of *R. toxicus* and other *Rathayibacter* species were obtained from independent culture collections (Table 1). The closely related species *R. agropyri*, *R. rathayi*, *R. iranicus*, and *R. tritici* were included in the exclusivity panel (Table 1). Strains were grown on 523M medium [3] and genomic DNA was extracted using the DNeasy Blood & Tissue Kit (Qiagen, Valencia, CA) following the manufacturer’s instructions. DNA from plant samples was isolated using Plant Material Lysis Kits (OptiGene, West Sussex, UK) as per the manufacturer’s instructions. DNA concentration was determined using a NanoDrop 1000 spectrophotometer (Thermo Fisher Scientific Inc., Worcester, MA, USA). 

### 2.2. Gene Selection and RPA Primer and Probe Design

**Figure 1:**
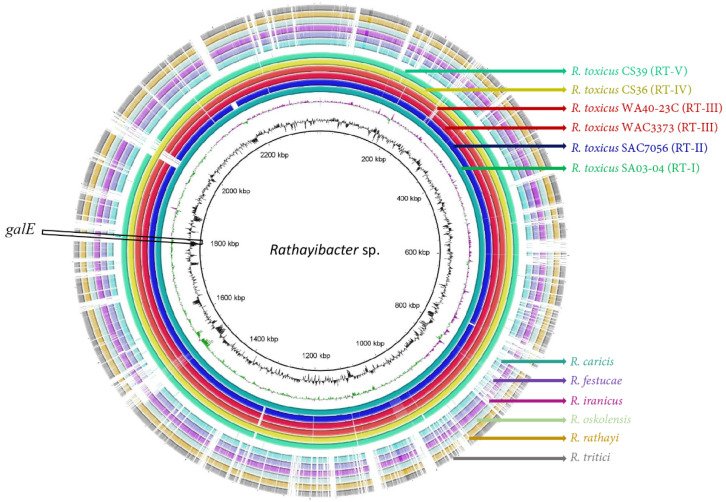
A ring image (BLAST Ring Image Generator) was used to locate the target UDP-glucose 4-epimerase (*galE*) gene region. Six genomes of *Rathayibacter toxicus* representing all known genetic populations RT-I to RT-V (respectively, RT-I: SA03-04, RT-II: SAC7056, RT-III: WAC3373 and WA40-23C, RT-IV: CS36, and RT-V: CS39) along with other *Rathayibacter* species, including *R. tritici* (CP015515)*, R. rathayi* (CP028129)*, R. iranicus* (CP028130)*, R. caricis* (GCF_003044275.1)*, R. festucae* (CP028137)*,* and *R. oskolensis* (GCF_900177245.1); from the center out: genome coordinates (kbp), GC content (black), GC skew (purple/green). The remaining rings show the BLASTn comparison of the 11 complete genomes as labelled. *Rathayibacter toxicus* population RT-I strain SA03-04 was used as the reference genome for comparison with the other genomes and to generate the ring image using BRIGS.

Whole-genome sequence analysis was performed to identify unique gene regions of *R. toxicus* to specifically detect this pathogen [31]. Six genomes of *R. toxicus* representing the known genetic populations RT-I to RT-V (respectively, RT-I: SA03-04, RT-II: SAC7056, RT-III: WAC3373 and WA40-23C, RT-IV: CS36, and RT-V: CS39) along with the genomes of six other *Rathayibacter* species, including the *R. tritici* strain NCPPB1953 (GenBank: CP015515)*, R. rathayi* strain DSM7485 (GenBank: CP028129)*, R. iranicus* strain NCCPB2253 (GenBank: CP028130.1)*, R. caricis* strain DSM15933 (GenBank: GCF_003044275.1)*, R. festucae* strain DSM15932 (GenBank: CP028137)*,* and *R. oskolensis* strain VKM Ac-2121 (GenBank: GCF_900177245.1); the genomes of other *Rathayibacter* species were retrieved from the NCBI GenBank database (reference numbers provided). Alignment of the genomes was performed using progressiveMauve 2.4.0 [32] and generated locally collinear blocks (LCBs) were individually screened with Geneious Prime to locate the candidate gene, UDP-glucose 4-epimerase (*galE*). The genome of *R. toxicus* SA03-04 was used as a reference to generate a comparative genome ring image using the BLAST Ring Image Generator (BRIGS) [33]; the NCBI GenBank ”ncbi-blast 2.6.0+” database was used to compare and generate BRIG images (Figure 1). The RPA primers and probe were designed using the *galE* target gene region sequence unique to *R. toxicus*. Specificity of the primers and probe was assessed in silico against the genomes of *R. tritici, R. rathayi, R. iranicus, R. caricis, R. festucae, R. oskolensis*, and *R. tanaceti*. The primers and probe sequences were also blasted against the NCBI GenBank database; the only similar sequences found were in *R. toxicus* (data not shown). To compare the two technologies (RPA and PCR), the same *galE* target region was used to design endpoint PCR primers using Primer3 [34] following the protocol of Arif and Ochoa-Corona [35], and thermodynamic parameters were evaluated [36]. The Rtox primers (Table 2) were used for the endpoint PCR assays while the RT-RPA primers and probe (Table 2) were used for the *R. toxicus* RPA assay.

For the plant host control, multiple ITS sequences of the main host, *Lolium rigidum*, as well other grass hosts of *R. toxicus* (*Agrostis stolonifera*, *A. capillaris*, *Polypogon monspeliensis*, *Avena sativa*, *Vulpia myuros*, and *Phalaris minor*) were retrieved from the NCBI GenBank database and aligned. The RPA primers and probe were designed targeting portions of a conserved region of the ITS gene. The IC-RPA primers and probe (Table 2) were used for the RPA assay for the host plant *L. rigidum* internal control.

### 2.3. RPA, Endpoint PCR, and Artificial Positive Control

TwistDx nfo kit (TwistDx Limited, Maidenhead SL6 4XE, UK) was used for DNA amplification following the manufacturer’s protocols. A 50 μL reaction contained 29.5 μL of rehydration buffer, 0.6 μL of (10 μM) of probe, 2.1 μL (10 μM) of each forward and biotin-labeled reverse primer (Table 2), 1 μL of purified DNA template from *R. toxicus* culture or 5 μL of plant tissue DNA isolated using Plant Material Lysis Kits, 2.5 μL of magnesium acetate (280 mM) to activate the RPA reaction, and nuclease-free water to complete the reaction volume (12.2 μL or 8.2 μL). All RPA assays were performed at 37 °C for 30 min in a Genie II (OptiGene) and each run was conducted with a positive control and a non-template control. After amplification, 2 μL of amplified product was mixed with 400 μL of nuclease-free water plus 100 μL of buffer (Milenia Biotec, Giessen, Germany). A Lateral Flow Device (Milenia HybriDetect 1; single analyte detection) was vertically inserted into the dilution mix and left for 1–2 min. A similar protocol was followed for the host control RPA assay using the relevant primers and probe for *L. rigidum* (Table 2). Biotin was added at the 5′ position of the RPA reverse primers to facilitate LFD detection of amplicons. FAM is a fluorescent dye incorporated for the LFD detection of RPA amplicons by gold particle-bound antibodies. 

Endpoint PCR primers targeting the same gene were also designed and validated (Table 2). The GoTaq Green Master Mix (Promega, Madison, WI) was used for PCR amplification. The reaction components were as follows: a 25 μL reaction contained 12.5 μL of GoTaq Green Master Mix, 1 μL (5 μM) of each forward and reverse primer, 1 μL of DNA template, and 9.5 μL of nuclease free water. The conditions were: initial denaturation at 94 °C for 2 min, followed by 35 cycles of 94 °C for 20 s, 56 °C for 30 s, and 72 °C for 30 s, and a final extension of 3 min at 72 °C. PCR amplifications were performed in a PTC-200 Peltier thermal cycler and DNA Engine (Bio-Rad, Hercules, CA, USA). Amplicons were separated using agarose gel (1.5%) electrophoresis in 1X TAE buffer, stained with 0.4 μg/mL ethidium bromide, and amplicons were visualized under a UV illuminator.

A positive control plasmid was developed by inserting the RPA primer/probe sequences into pUCIDT-AMP (synthesized by IDT). The total size of the plasmid with insert was 3183 bp. Using the appropriate primers and probe, the positive control plasmid produced amplicons of 151 bp and 169 bp for RPA and PCR, respectively.

### 2.4. RPA and Endpoint PCR Specificity and Sensitivity Assays

The specificity of the developed RPA primers and probes was evaluated using an inclusivity panel comprised of 67 strains from the five known genetic populations of *R. toxicus* collected during the last four decades—and the exclusivity panel comprised of multiple strains of *R. tritici, R. agropyri, R. iranicus, R. rathayi, Dietzia cinnamea*, and *Clavibacter nebraskensis* (Table 1). The specificity of the endpoint PCR was also evaluated with the same inclusivity and exclusivity panels. The specificity was also evaluated by performing an assay with the DNA isolated from the field samples (SA03, SA08, SA19, SA70, WA06, WA08, WA41, WA61, WA64, WA68, and WA69) collected from South Australia and Western Australia in 2014.

The limit of detection for the RPA assay, with and without host background, was determined using tenfold serial dilutions—1 ng to 1 ag—of *R. toxicus* genomic DNA. The spiked assay was performed by adding 5 µL of crude host DNA into each tenfold serially diluted sensitivity reaction. A No-Template Control (NTC) was included to confirm the reliability and accuracy of the assay. The endpoint PCR sensitivity and spiked sensitivity assays were also performed using the same dilutions to compare the results. RPA and endpoint PCR sensitivity assays were also performed with tenfold serial dilutions of plasmid DNA (positive control) containing the target primer and probe sequences.

### 2.5. Hand-Held RPA Amplification

To preclude the need for a heat block in the field in order to maintain a constant reaction temperature, the efficacy of running the RPA reaction while holding the reaction tubes in a closed hand was evaluated for performance and specificity. Four reactions were performed with DNA from two *R. toxicus* strains (SA08-08 and WAC7056), one *R. rathayi* strain (ICMP 2579), and one NTC. The RPA assay components were used as mentioned above.

### 2.6. In-Field Performance

The in-field performance of the RPA assay was tested at different locations in South Australia previously determined to be positive sites for *R. toxicus* [14,30]. At each field site, multiple plant samples were collected from different areas and the RPA assay run for the detection of *R. toxicus*. A Plant Material Lysis Kit was used for DNA extraction (crude DNA) following the manufacturer’s instructions (OptiGene), eliminating the need for standard lab equipment [23]. In addition to the *R. toxicus* RPA assay, each sample was tested with the host control RPA assay to confirm successful DNA preparation. The RPA protocol was followed as mentioned above except 5 μL of DNA extract was added in each reaction instead of 1 μL. Each sample was also tested with an *R. toxicus*-specific LAMP assay [12] to compare performance in the field. 

### 2.7. Comparative Plant Inhibitory Effect

Extracts of rose leaf tissue, known for PCR inhibitors [29], and ryegrass seed, a common host for *R. toxicus*, were used to evaluate potential inhibitory effects from plant tissue components on the RPA assay. A 100 mg sample of rose leaves or annual ryegrass seeds was placed into a 1.5 mL Eppendorf tube with 1.0 mL of sterile water and macerated using a small pestle. Three techniques with four different chemistries were used (endpoint PCR, LAMP with no kit, LAMP with OptiGene kit, and RPA) with four reactions for each chemistry (1—*R. toxicus* purified genomic DNA; 2—*R. toxicus* purified genomic DNA + 2 µL rose leave extract; 3—*R. toxicus* purified genomic DNA + 2 µL annual rye grass seed extract; 4—NTC/water). 

## 3. Results

### 3.1. Primer and Probe Design and in Silico Specificity

Representative genomes of all five genetic populations of *R. toxicus* (SA03-04, SAC7056, WAC3373, WA40-23C, CS36, and CS39) along with other *Rathayibacter* species, including *R. tritici, R. rathayi, R. iranicus, R. caricis, R. festucae, R. oskolensis*, and *R. tanaceti*, were evaluated to identify taxon-specific diagnostic markers in *R. toxicus* (Figure 1). Based on the genomes of different populations of *R. toxicus* [11,30,31] and other *Rathayibacter* species, *galE* was selected for primers and probe design. The BLASTn outcomes showed 100% query coverage and 100% identity only with *R. toxicus* genomes when the primers and probe were evaluated using NCBI GenBank database; no 100% matching with any other species was observed. The primers/probes were also evaluated with 18 *R. toxicus* genomes present in our *in-house* database [31]—the selected signature region was highly conserved among all the genomes and showed 100% identity with all *R. toxicus*-specific primers and probe. Primers were thermodynamically competent to obtain the highest sensitivity. 

### 3.2. Specificity Assays

**Figure 2:**
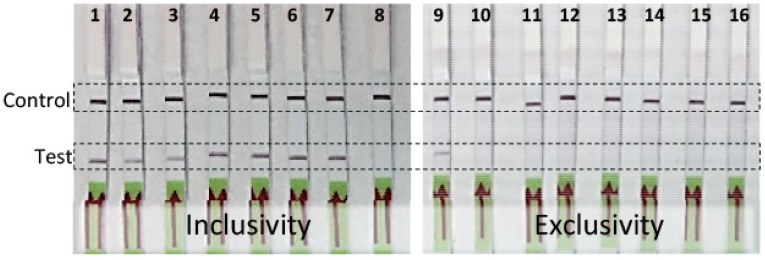
Validation of a *Rathayibacter toxicus*-specific recombinase polymerase amplification (RPA) assay with inclusivity and exclusivity panels; panel details are provided in Table 1. RPA amplification products were visualized with lateral flow strips. The upper bands on the lateral flow strips are the internal lateral flow strip control, the lower bands are the RPA reaction products. Lanes 1–8 (inclusivity panel results): 1—*R. toxicus* (SA03-04, RT-I); 2—*R. toxicus* (SA08-08, RT-I); 3—*R. toxicus* (SA19-02, RT-I); 4—*R. toxicus* (CS14, RT-II); 5—*R. toxicus* (CS28, RT-III); 6—*R. toxicus* (CS33, RT-II); 7—*R. toxicus* (CS34, RT-II); 8—water (non-template control). Lanes 9–16 (exclusivity panel results): 9—*R. toxicus* (SA08-08, positive control); 10—*R. tritici* (WAC7055); 11—*R. agropyri* (WAC9621); 12—*R. iranicus* (ICMP 3494); 13—*R. rathayi* (ICMP 2574); 14—*Dietzia cinnamea* (SA03-14M); 15—*Clavibacter michiganensis* subsp. *nebraskensis* (Cmn); 16—water (non-template control).

**Figure 3:**
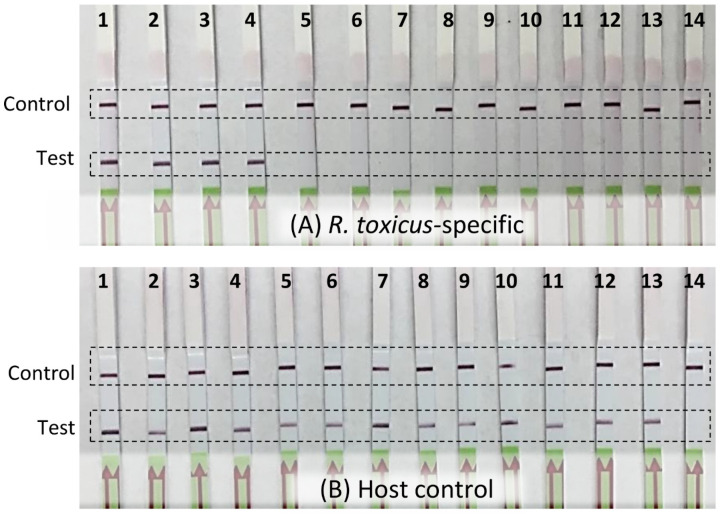
Detection of *Rathayibacter toxicus* from annual ryegrass samples using a recombinase polymerase amplification (RPA) assay. Annual ryegrass samples were collected in 2014 at multiple locations in South Australia and Western Australia; DNA was isolated and RPA assays were performed using (**A**) *R. toxicus*-specific primers/probe and (**B**) host plant-specific primers/probe. Lane A1: positive control (*R. toxicus* strain SA03-04); lane B1: positive control (annual ryegrass DNA); lanes A2–A12 and B2–B12: field sample SA03; SA08; SA19; SA70; WA06; WA08; WA41; WA61; WA64; WA68; WA69; lanes A13 and B13: negative control (host DNA); lanes A14 and B14: water (non-template control).

**Figure 4:**
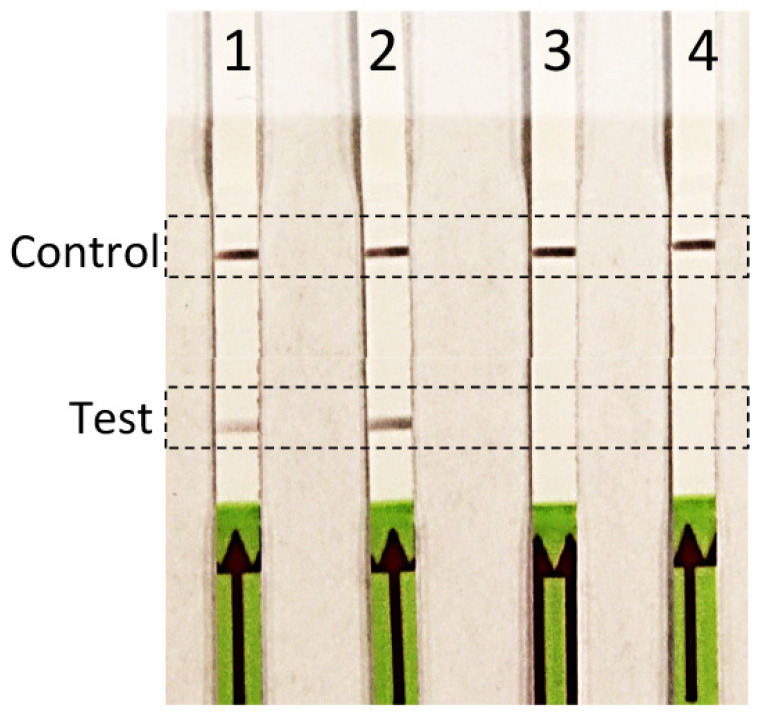
Verification of efficacy for hand-held *Rathayibacter toxicus*-specific recombinase polymerase amplification (RPA) assay; reaction tubes were enclosed in the palm of the hand (no heat block was required). Four RPA reactions were performed with genomic DNA from two *R. toxicus* strains (lane 1: SA08-08 and lane 2: WAC7056), one *R. rathayi* strain (lane 3: ICMP 2579), and water, a non-template control (lane 4).

RPA assay specificity was determined with an inclusivity panel comprised of 67 strains of *R. toxicus* from the five known genetic populations, RT-I, RT-II, RT-III, RT-IV, and RT-V. RPA and endpoint PCR assays were performed and the results compared; no discrepancy between the two assays was observed (Table 1). No amplification was observed with any of the strains in the exclusivity panel that was comprised of strains representing the other *Rathayibacter* species: *R. tritici, R. agropyri, R. iranicus,* and *R. rathayi* (Table 1; Figure 2). The annual ryegrass samples from 11 sites (SA03; SA08; SA19; SA70; WA06; WA08; WA41; WA61; WA64; WA68; WA69) from South Australia and Western Australia were tested for *R. toxicus*; three sites (SA03, SA08, and SA19) were positive for *R. toxicus* (Figure 3A; previously confirmed sites for *R. toxicus* infection) [30]. The samples were also tested with the host RPA assay (Figure 3B). Endpoint PCR was also tested with *R. toxicus* specific primers; results were concordant. The specificity was also tested by incubating the RPA reactions in the closed palm of the hand—no false positive or false negative results were obtained (Figure 4). Overall, the developed *R. toxicus*-specific RPA assay was highly specific and robust. 

### 3.3. Sensitivity Assays

**Figure 5:**
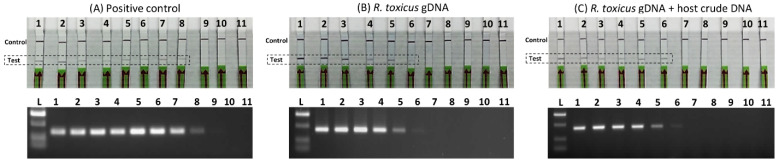
Sensitivity and spiked sensitivity validation of the *Rathayibacter toxicus*-specific recombinase polymerase amplification (RPA) assay. The RPA assay results were compared to the results of a *R. toxicus*-specific endpoint PCR assay. Tenfold serial dilutions (1 ng to 1 ag) were used to perform the assays; upper images are the RPA results and lower images are the endpoint PCR results. (**A**) RPA and endpoint PCR assays with tenfold dilution of positive control (plasmid DNA-carrying primers and probe target sequences); (**B**) RPA and endpoint PCR assays with tenfold serially diluted *R. toxicus* genomic DNA; (**C**) RPA and endpoint PCR assays with tenfold serially diluted *R. toxicus* genomic DNA plus 5 µL of crude host (ryegrass) DNA.

The sensitivities of the *R. toxicus*-specific RPA assay and the endpoint PCR assay were compared; the endpoint PCR and RPA primers were designed from the same genomic region to more directly compare the performance of the RPA assay. Three sensitivity assays were performed (Figure 5): (A) tenfold dilution of the positive control (plasmid DNA carrying primers and probe target sequences; (B) tenfold serially diluted *R. toxicus* genomic DNA; and (C) tenfold serially diluted *R. toxicus* genomic DNA plus 5 µL of crude host (ryegrass) DNA. The detection limits were 10 fg (approximately 4 *R. toxicus* cells) for the RPA assay and 100 fg (approximately 40 *R. toxicus* cells) for the endpoint PCR assays. However, with the endpoint PCR assay, a faint band was observed with 10 fg. The sensitivity with plasmid DNA was ~100 ag; this higher sensitivity may have been due to the smaller size of the plasmid (total size ~3.1 kb including target) compared to *R. toxicus* genomic DNA of about ~2.3 Mb (Figure 5), which resulted in higher copy numbers in the same amount of DNA. Overall, the RPA assay was highly sensitive, thus reducing the probability of false negatives in cases of low (latent) infection levels.

### 3.4. Plant Inhibitory Effect

**Figure 6:**
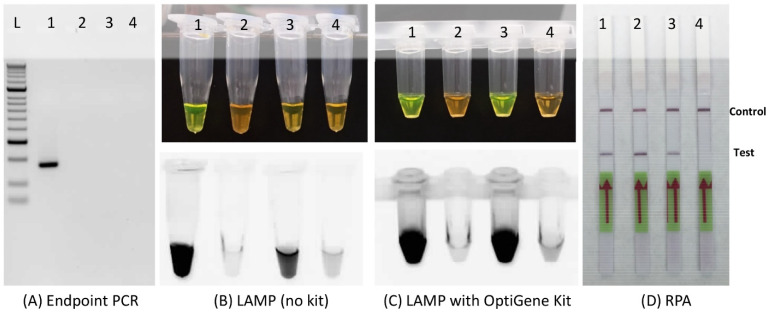
Effects of plant inhibitors on the detection of *Rathayibacter toxicus* using (**A**) endpoint PCR, (**B**,**C**) loop-mediated isothermal amplification (LAMP), and (**D**) recombinase polymerase amplification; detection assays based on the three technologies using four chemistries were compared. (**A**–**D**) Lane 1—purified *R. toxicus* genomic DNA; lane 2—purified *R. toxicus* genomic DNA plus 1 µL of rose leaf extract; lane 3—purified *R. toxicus* genomic DNA plus 1 µL of ryegrass seed extract; and lane 4—water (non-template control). (**B**,**C**) Upper pictures were taken in daylight with a regular camera; lower pictures were taken under UV light in a GelDoc system. The RPA assay was unaffected by either the rose tissue extract or the ryegrass seed extract whereas the endpoint PCR was inhibited by both. The LAMP assay was inhibited by the rose tissue extract and sensitive to the ryegrass seed extract depending on the reagents used.

The developed RPA assay was also compared for resistance to plant inhibitors. Three commonly used techniques based on four chemistries were compared. The *R. toxicus* DNA was spiked with rose extract or ryegrass seed extract. Endpoint PCR was very sensitive to inhibitors present in both the rose and ryegrass plant extracts. The results clearly indicated that RPA was not affected by inhibitors from either plant extracts (Figure 6). The LAMP assay performed using the OptiGene kit was more resistant to plant inhibitors compared to the LAMP reaction with no kit (individual reagents were used to prepare the reaction mix in place of the master mix kit). The LAMP assays were sensitive to reaction inhibitors in the rose extract but less so to the ryegrass extracts (Figure 6).

### 3.5. On-Site Detection of R. toxicus

**Figure 7:**
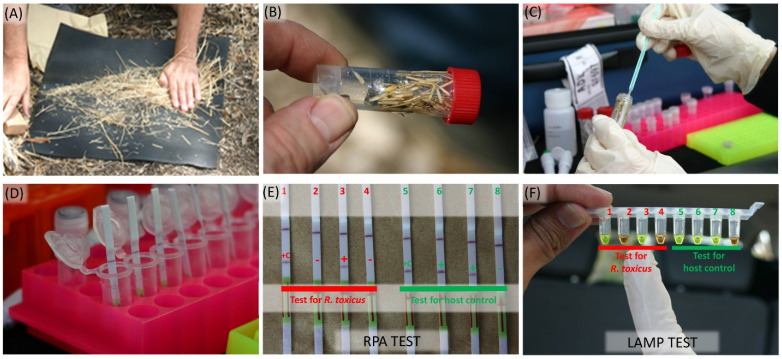
In-field detection of *Rathayibacter toxicus* from cultivated and non-cultivated annual ryegrass (*Lolium rigidum*) plants using a validated recombinase polymerase amplification (RPA) assay and a *R. toxicus*-specific loop mediated isothermal amplification (LAMP) assay: (**A**) annual ryegrass seeds were separated from plants; (**B**) samples were processed (OptiGene Plant Lysis Kit); (**C**) seed extract (one loopful, ca. 10 µL) was transferred into a 1.0 mL OptiGene tube with buffer; (**D**) post-reaction, lateral flow strips (LFDs) were placed in a 500 µL buffer (400 µL water + 100 µL buffer provided with the kit) containing 2 µL of RPA amplified product; (**E**) RPA reaction products visualized with LFD; (**F**) RPA reaction products visualized with LAMP assay—there was no discrepancy between the RPA and LAMP results.

The performance and specificity of the RPA assay was tested at two known *R. toxicus* positive field sites in South Australia. At each site, annual ryegrass samples were collected, and the RPA assay performed on-site for the detection of *R. toxicus* (Figure 7). All samples from each area were positive when tested with the host control ITS RPA assay, indicating that the DNA preparation was successful for each sample. The *R. toxicus* RPA assay detected *R. toxicus*-positive samples at both sites. Not all samples were positive; *R. toxicus* is typically patchy in distribution. The accuracy of the RPA results was cross-confirmed using a LAMP assay [12] on the same samples; both assays yielded the same results (Figure 7). This also supports the observation that the LAMP assay was not negatively affected by potential inhibitors in the ryegrass. On-site, the assays were consistently performed by multiple operators with no false positives and negatives in any tests.

## 4. Discussion

Comparative genomics analysis to identify distinctive genomic regions of high diagnostic value has now become a common approach to support the design of robust and highly specific assays for genus-, species-, and strain-level discrimination [14,24,26,31,36,37]. Designing primers and probes from unique genomic regions enhances assay robustness and reduces the probability of non-specific amplification. In this study, we designed, developed, and field tested a reliable, sensitive, and rapid field-deployable recombinase polymerase assay and an endpoint PCR assay for the specific detection of the Select Agent *R. toxicus* based on the signature gene region within *galE*, present only in *R. toxicus*. All primers and probes designed to be used in the RPA and endpoint PCR assays showed 100% identity with *R. toxicus* genomes when aligned using BLASTn with the NCBI GenBank database. The primers and probes were thermodynamically competent to achieve high sensitivity [35], thus minimizing the likelihood of false negatives in the case of latent infections. 

The application of isothermal amplification technologies is increasing in both lab and field settings. LAMP assays have a moderately high temperature of ~65 °C for amplification of the target genome region which requires some type of thermal device [24]. One of the main advantages of RPA over other amplification technologies is the relatively low reaction temperature of 37–42 °C and a rapid reaction time of 15–30 min with high accuracy [26,38]. The TwistDx nfo kit is available in lyophilized form and can easily be transported and used in field settings without the need for sophisticated instruments. The *R. toxicus* RPA assay showed high specificity when tested on broad and extensive inclusivity (strains from all reported populations of *R. toxicus*) and exclusivity (strains from several *Rathayibacter* spp.) panels (Table 1). The RPA inclusivity results were comparable with the endpoint PCR results; no discrepancy was observed. The high specificity of genome-informed RPA assays for the detection of other target organisms was also reported by Ahmed et al. [26] and Boluk et al. [27]; both used a similar methodology (RPA amplification using TwistDx nfo coupled with LFDs). The developed assays also accurately detected the target genome sequence when reactions were performed in a closed fist (Figure 4), further demonstrating the lack of a requirement for additional equipment in the field.

One of the most important characteristics of a diagnostic assay is high sensitivity, the ability to detect the target signal at low concentrations. From a biosecurity perspective, this is critical to reduce the likelihood of false negative assay results that may lead to the introduction of potentially harmful organisms into new environments. The *R. toxicus* RPA assay developed in this study detected target DNA to 10 fg with and without a background of host genomic DNA; RPA assay sensitivity was approximately tenfold higher than the endpoint PCR sensitivity. Two additional challenges to successful detection of a target DNA sequence include target dilution by host DNA and the presence of compounds that inhibit the polymerases that amplify the target sequence. Tolerance of amplification inhibitors present in the sample matrix is a critical attribute for diagnostic assays. Amplification inhibitors are common in tissues of several plant species. No adverse effects were observed when 5 µL of host crude DNA were added into each RPA reaction containing tenfold serially diluted *R. toxicus* DNA and, importantly, there was no evidence of amplification inhibition caused by plant-derived compounds. Similarly, Ahmed et al. [26] and Boluk et al. [27] reported that RPA was not affected by plant inhibitors, even when the plant tissues were macerated in TE buffer and the extract used for target amplification. In this study, no inhibition was observed when rose tissue extract or ryegrass seed extract was added into RPA reaction mixtures; rose tissue and plant seed are known for high PCR inhibition [29].

Diagnostic results inform the decision making that supports response to incursions of potentially harmful organisms. Confidence in the performance of diagnostic assays is, in part, a function of the inclusion of proper and verified positive and negative controls in assay execution. Designing positive controls by inserting primer sequences into a plasmid is an easy and effective method to generate and maintain positive controls based on multiple primer sets [28], thus providing direct assessment data for determination of assay quality control, accuracy, and reliability. In this study, we designed and synthesized a positive control plasmid containing target sequences for the *R. toxicus* RPA primers and probe. This plasmid was used as a positive control as well as to accurately determine assay sensitivity. The developed plant host DNA control also enhanced the reliability of the assays. The likelihood of either false positive or false negative assay results was greatly reduced, increasing the confidence in the results that ultimately may support biosecurity surveillance and response.

The developed *R. toxicus*-specific RPA assay coupled with LFDs was highly specific and detected the target in 30 min or less, in the lab and in the field. The assay was not affected by plant inhibitors, thereby precluding the need for DNA isolation (often a challenge in some field settings); a crude DNA preparation using the OptiGene Plant DNA isolation kit or tissue macerated using TE buffer was sufficient. The following characteristics made this assay fully field-deployable: no DNA isolation required, lyophilized assay reagents, LFD-based visualization, no equipment required, no detectable effect of sample matrix inhibitors, and robust and rapid performance. Applications for the developed RPA assay include routine diagnostics, biosecurity surveillance, microbial forensics, and disease epidemiology and management.

## 5. Conclusions

Early detection and accurate identification of potentially harmful organisms, including plant pathogens, is essential for successful prevention and mitigation outcomes. Nucleic acid-based technologies (NATs) based on the polymerase chain reaction (PCR) have provided the specificity and sensitivity required for effective detection and identification in the lab. However, PCR-based methods require sophisticated lab equipment and are most often not conducive for *on-site* detection of plant pathogens in environmental or agricultural settings. Applications using isothermal nucleic acid amplification methods are increasing due to ease of use and rapid performance. RPA is gaining in popularity because of its unique characteristics including low reaction temperature (37 °C–39 °C) for amplification and insensitivity to plant inhibitors. Here, we developed a field-deployable RPA assay coupled with LFDs that can detect and identify *R. toxicus* from bacterial culture and infected plant tissues. The developed assay was tested with extensive inclusivity and exclusivity panels to confirm high specificity and accuracy. The assay is highly insensitive to plant inhibitors and does not require any DNA isolation. Moreover, DNA amplification can be obtained in a closed-hand palm at body temperature without any non-specific outcome. The developed method described here provides a framework to develop and validate field-deployable RPA assays for other plant pathogens.

## Figures and Tables

**Table 1:** Strains included in inclusivity and exclusivity panels to confirm specificity of the developed PCR and recombinase polymerase assays to specifically detect *Rathayibacter toxicus*.

Species	Strain Name	Year	Host	Geographical Location	Results PCR	Results RPA
*Rathayibacter toxicus*	SA03-02	2014	ARG	Corny Point, SA	+	+
*R. toxicus*	SA03-03	2014	ARG	Corny Point, SA	+	+
*R. toxicus*	SA03-04	2014	ARG	Corny Point, SA	+	+
*R. toxicus*	SA03-08	2014	ARG	Corny Point, SA	+	+
*R. toxicus*	SA03-14	2014	ARG	Corny Point, SA	+	+
*R. toxicus*	SA03-15	2014	ARG	Corny Point, SA	+	+
*R. toxicus*	SA03-16	2014	ARG	Corny Point, SA	+	+
*R. toxicus*	SA03-17	2014	ARG	Corny Point, SA	+	+
*R. toxicus*	SA03-18	2014	ARG	Corny Point, SA	+	+
*R. toxicus*	SA03-19	2014	ARG	Corny Point, SA	+	+
*R. toxicus*	SA03-20	2014	ARG	Corny Point, SA	+	+
*R. toxicus*	SA03-21	2014	ARG	Corny Point, SA	+	+
*R. toxicus*	SA03-22	2014	ARG	Corny Point, SA	+	+
*R. toxicus*	SA03-23	2014	ARG	Corny Point, SA	+	+
*R. toxicus*	SA03-24	2014	ARG	Corny Point, SA	+	+
*R. toxicus*	SA03-25	2014	ARG	Corny Point, SA	+	+
*R. toxicus*	SA03-26	2014	ARG	Corny Point, SA	+	+
*R. toxicus*	SA03-27	2014	ARG	Corny Point, SA	+	+
*R. toxicus*	SA03-28	2014	ARG	Corny Point, SA	+	+
*R. toxicus*	SA08-03	2014	ARG	Lake Sunday, SA	+	+
*R. toxicus*	SA08-07	2014	ARG	Lake Sunday, SA	+	+
*R. toxicus*	SA08-08	2014	ARG	Lake Sunday, SA	+	+
*R. toxicus*	SA08-09	2014	ARG	Lake Sunday, SA	+	+
*R. toxicus*	SA08-11	2014	ARG	Lake Sunday, SA	+	+
*R. toxicus*	SA08-13	2014	ARG	Lake Sunday, SA	+	+
*R. toxicus*	SA08-16	2014	ARG	Lake Sunday, SA	+	+
*R. toxicus*	SA19-02	2013	ARG	Yorketown, SA	+	+
*R. toxicus*	SA19-03	2013	ARG	Yorketown, SA	+	+
*R. toxicus*	SA19-04	2013	ARG	Yorketown, SA	+	+
*R. toxicus*	SA19-05	2013	ARG	Yorketown, SA	+	+
*R. toxicus*	SA19-06	2013	ARG	Yorketown, SA	+	+
*R. toxicus*	SA19-07	2013	ARG	Yorketown, SA	+	+
*R. toxicus*	SA19-08	2013	ARG	Yorketown, SA	+	+
*R. toxicus*	SA19-09	2013	ARG	Yorketown, SA	+	+
*R. toxicus*	SA19-10	2013	ARG	Yorketown, SA	+	+
*R. toxicus*	SA19-11	2013	ARG	Yorketown, SA	+	+
*R. toxicus*	SA19-12	2013	ARG	Yorketown, SA	+	+
*R. toxicus*	SA19-13	2013	ARG	Yorketown, SA	+	+
*R. toxicus*	SA19-14	2013	ARG	Yorketown, SA	+	+
*R. toxicus*	SAC3368	1981	ARG	SA	+	+
*R. toxicus*	SAC3387	1981	ARG	SA	+	+
*R. toxicus*	SAC7056	1983	ARG	Murray Bridge, SA	+	+
*R. toxicus*	WAC3371	1978	LCG	Gnowangerup, WA	+	+
*R. toxicus*	WAC3372	1978	BO	Gnowangerup, WA	+	+
*R. toxicus*	WAC3373	1978	PG	Gnowangerup, WA	+	+
*R. toxicus*	WAC3396	1980	Oat	Gnowangerup, WA	+	+
*R. toxicus*	CS1			SA	+	+
*R. toxicus*	CS3		ARG	WA	+	+
*R. toxicus*	CS28	1978	ARG	WA	+	+
*R. toxicus*	CS29	1981	ARG	WA	+	+
*R. toxicus*	CS30	1980	Oat	WA	+	+
*R. toxicus*	CS31	1981	*Phalaris* sp.	WA	+	+
*R. toxicus*	CS32	1981	DC	WA	+	+
*R. toxicus*	CS33	1984	ARG	SA	+	+
*R. toxicus*	CS34	1983	ARG	SA	+	+
*R. toxicus*	CS36	1990	PBG	Gongolgon, NSW	+	+
*R. toxicus*	CS38	1990	ABG	Lucindale, SA	+	+
*R. toxicus*	CS39	1990	ABG	Lucindale, SA	+	+
*R. toxicus*	WA40-18A	2015	ARG	WA	+	+
*R. toxicus*	WA40-18B	2015	ARG	WA	+	+
*R. toxicus*	WA40-20A	2015	ARG	WA	+	+
*R. toxicus*	WA40-20B	2015	ARG	WA	+	+
*R. toxicus*	WA40-21A	2015	ARG	WA	+	+
*R. toxicus*	WA40-21B	2015	ARG	WA	+	+
*R. toxicus*	WA40-23A	2015	ARG	WA	+	+
*R. toxicus*	WA40-23B	2015	ARG	WA	+	+
*R. toxicus*	WA40-23C	2015	ARG	WA	+	+
*R. tritici*	WAC7055	1991	Whaet	Carnamah, WA	Negative	Negative
*R. tritici*	WAC9601	-	RG		Negative	Negative
*R. tritici*	WAC9602	-	RG		Negative	Negative
*R. rathayi*	ICMP 2574	1968	DG	New Zealand	Negative	Negative
*R. rathayi*	WAC3369	-	ARG	WA	Negative	Negative
*R. rathayi*	ICMP 2579	-	DG	United Kingdom	Negative	Negative
*R. iranicus*	ICMP 13126	1994	Wheat	Iran	Negative	Negative
*R. iranicus*	ICMP 13127	1994	Wheat	Iran	Negative	Negative
*R. iranicus*	ICMP 12831	1994	Wheat	Iran	Negative	Negative
*R. iranicus*	ICMP 3496	-	Wheat	-	Negative	Negative
*R. agropyri*	WAC9620		RG		Negative	Negative
*R. agropyri*	WAC9621				Negative	Negative
*R. agropyri*	WAC9622				Negative	Negative
*R. agropyri*	WAC9594		RG		Negative	Negative
*Dietzia cinnamea*	SA03-14M	2014	ARG	Corny Point, SA	Negative	Negative
*Clavibacter nebraskensis*	Cmn	-	-	-	Negative	Negative
Soil	Non-infested soil	-	-	-	Negative	Negative
Host	Healthy ryegrass	-	-	-	Negative	Negative

PBG—Pacific bent grass (*Agrostis avenacea*); ABG—annual beard grass (*Polypogon monspeliensis*); ARG—annual ryegrass (*Lolium rigidum*); RG—ryegrass (*Lolium* sp.); LCG—lesser canary grass (*Phalaris minor*); BO—black oat (*Avena fatua*); PG—paradoxa grass (*Phalaris paradoxa*); DC—*Danthonia caespitosa*; Oat (*Avena sativa*); DG—*Dactylis glomerata*. NSW—New South Wales; SA—South Australia; WA—Western Australia.

**Table 2:** Characteristics of the primers and probes used in the endpoint PCR (Rtox-F1 and Rtox-R1) and recombinase polymerase assays for the detection of *Rathayibacter toxicus* (RT-RPA-F, RT-RPA-R*, and RT-RPA-P) and its host *Lolium rigidum* (host control: IC-RPA-F, IC-RPA-R*, and IC-RPA-P.

Name	Sequence (5′-3′)	GC %	Length (bp)	Amplicon Size (bp)
Rtox-F1	GACAATTTATCGACGGGTGA	45.0	20	170
Rtox-R1	AGCGGCTCGCTTACAGATT	52.6	19	
RT-RPA-F	AAGTGACGGTGATCGACAATTTATCGACGGGTGAC	49	35	189
RT-RPA-R	* BiosG-ATATCAGCGGCTCGCTTACAGATTCTTTGACCGAC	49	35	
RT-RPA-P	** FAM-CAGATATTTCGGAAGTTGATCACATAGTCG-dSpacer-CGGAACTCAGTGGTGTTTCT-SpacerC3	50	44	
IC-RPA-F	TAATCCACACGACTCTCGGCAACGGATATCTC	32	50	123
IC-RPA-R	* BiosG-CAACTTGCGTTCAAAGACTCGATGGTTCGCG	31	52	
IC-RPA-P	** FAM-CTCGCATCGATGAAGAACGTAGCGAAATGC-dSpacer-ATACCTGGTGTGAATTGCA-SpacerC3	49	47	

* Biotin was added at the 5′ position of the RPA reverse primers to facilitate LFD detection of amplicons. ** FAM is a fluorescent dye incorporated for the LFD detection of RPA amplicons by gold particle-bound antibodies. The Rtox primers were used for the endpoint PCR assays. The RT-RPA and the IC-RPA primers and probe were used for the recombinase polymerase assays (RPA) for the *R. toxicus* target and the host plant *L. rigidum* internal control.

## Data Availability

All the sequences were downloaded from the NCBI GenBank database and are freely available.
